# Does Probiotic Consumption Enhance Wound Healing? A Systematic Review

**DOI:** 10.3390/nu14010111

**Published:** 2021-12-27

**Authors:** Camille Togo, Ana Paula Zidorio, Vivian Gonçalves, Patrícia Botelho, Kenia de Carvalho, Eliane Dutra

**Affiliations:** 1 Graduate Program in Human Nutrition, Faculty of Health Sciences, University of Brasilia, Campus Universitario Darcy Ribeiro, Brasilia 70910-900, Brazil; anacaio@unb.br (A.P.Z.); patriciabotelho@unb.br (P.B.); kenia@unb.br (K.d.C.); elidutra@unb.br (E.D.); 2 Clinical Nutrition Unit, University Hospital of Brasilia, Brasilia 70840-901, Brazil; 3 Graduate Program in Public Health, Faculty of Health Sciences, University of Brasilia, Campus Universitario Darcy Ribeiro, Brasilia 70910-900, Brazil; vivian.goncalves@unb.br

**Keywords:** probiotics, skin wound, oral mucosal wound, wound healing, systematic review

## Abstract

The use of probiotics is one of the emerging lines of treatment for wound healing. This systematic review aimed to summarize currently available evidence on the effect of oral or enteral probiotic therapy on skin or oral mucosal wound healing in humans. To verify the developments in this field and the level of available scientific evidence, we applied a broad search strategy with no restrictions on wound type, target population, probiotic strain, or intervention protocol used. This review included seven studies involving 348 individuals. Four studies reported positive outcomes for healing improvement after probiotic therapy, and none of the studies reported adverse effects or a marked increase in wound healing time. The positive or neutral results observed do not generate strong evidence regarding the effectiveness of probiotics for wound healing. However, they suggest a promising field for future clinical research where the probiotic strains used, type of wounds, and target population are controlled for.

## 1. Introduction

Skin and mucosal wounds encompass a wide variety of injuries, including a surgical scar, pressure ulcer, extensive burn, and an open abdominal wound. Healing is an inherent process in all wounds, regardless of the cause. It is a systemic, dynamic, and expected process related to the general conditions of the organism [1], and can be negatively or positively influenced by local and systemic factors. The local factors that hinder wound healing include ischemia, infection, surgical technique, foreign body, and oedema [2], while systemic factors include vitamin deficiencies, malnutrition [3,4], and other conditions such as diabetes mellitus [5] and cardiovascular and respiratory diseases [1].

Wound care practices involve technical procedures, topical agents, and dressings, as well as holistic and systemic treatment, where the patient, and not just the wound, are observed [6]. An emerging treatment line for skin wounds and conditions is the use of probiotics, defined by the International Scientific Association of Probiotics and Prebiotics as “live microorganisms which when administered in adequate amounts confer a health benefit on the host” [7].

Probiotics have been extensively investigated, and their role in improving infections and intestinal healing is well-known [8,9]. Researchers have reported that probiotics may have other health-promoting effects beyond intestinal wellbeing [10,11,12], such as preventing recurrent urinary tract infections in women and reducing respiratory tract infections [13,14,15]. In addition, a narrative literature review reported the beneficial effects of oral administration and topical application of probiotics for the treatment of skin diseases [15].

Studies have been conducted in order to evaluate the use of probiotics to enhance wound healing. Yu et al. reviewed the usefulness of oral and topical probiotics for certain dermatological diseases. The authors suggested that these interventions could be effective in the treatment of certain inflammatory skin diseases, with a promising role in promoting wound healing and managing skin cancer [16]. However, the use of probiotics as a nutritional supplement to treat skin or mucosal wounds was not emphasized in their review [16]. Animal studies have demonstrated a positive effect of probiotics in reducing bacterial load and increasing tissue repair [17,18]. In addition, in vitro studies [8,19] have demonstrated a positive effect for probiotics on the recovery of structural elements of the skin.

Although in vitro and in vivo animal studies support the potential for probiotics to promote skin healing, it is of paramount importance to investigate the level of existing evidence reported in human studies. Therefore, the aim of this systematic review was to summarize currently available evidence on the effect of oral or enteral probiotic therapy on skin or oral mucosal wound healing in humans.

## 2. Materials and Methods

### 2.1. Protocol and Registration

This systematic review followed the Preferred Reporting Items for Systematic Reviews and Meta-Analyses (PRISMA) checklist [20] and the protocol was registered in the International Prospective Register of Systematic Reviews (PROSPERO) under the registration number CRD42020150682.

### 2.2. Eligibility Criteria

Randomised and non-randomised placebo-controlled trials that recruited people of all age groups and both sexes, with skin or oral mucosal wounds, receiving oral or enteral probiotic therapy with or without antibiotic treatment, and with healing assessment data, were included. Given that the preliminary searches identified few studies when limited to the type of wound, probiotic, or target population, we applied a broad search strategy to verify the scientific evidence level and evaluate the developments in this field. Reviews, congress abstracts, chapters of books, meeting proceedings, and studies without clear outcomes were not included. Furthermore, animal studies, in vitro studies, and studies with topical use of probiotics were excluded. There were no restrictions on the date, language, or publication status.

### 2.3. Information Sources and Search Strategies

The search strategy was developed based on the criteria recommended by the Peer Review of Electronic Search Strategies (PRESS) checklist [21] and submitted for revision by a researcher experienced in conducting systematic reviews. Detailed search strategies with adaptations of descriptors and term combinations were elaborated on according to the specificities of the following databases: Medline (through PubMed), Embase, Lilacs, Scopus, and Web of Science. A partial grey literature search was also conducted using the ProQuest database of theses and dissertations and Google Scholar, wherein the search was limited to the first 200 articles found. Finally, a search was performed of the clinical trials record database ClinicalTrials.gov (Table S1).

Rayyan QCRI software (Qatar Computing Research Institute^®^, Doha, Qatar) [22] was used to remove duplicates and facilitate screening of the identified records. All references were managed using Mendeley Desktop software (Version 1.19.8; Mendeley Ltd., London, UK).

### 2.4. Study Selection

The study selection process was conducted in two phases by two independent researchers. In phase one, articles were selected according to their titles and abstracts based on the inclusion criteria. Any disagreements were resolved by consensus. In phase two, the selected articles were read in their entirety, and those that met the inclusion criteria were included. A manual search of the reference lists of the selected articles was also performed.

### 2.5. Data Collection Process

Data extraction was also performed independently by two authors. Any disagreements were resolved by consensus. The following data were extracted from the selected articles and recorded in an electronic spreadsheet: country, aim and study design, subjects/patients, type of wounds, intervention protocol, healing assessments, outcome of interest, possible adverse effects of probiotic therapy, and whether probiotic therapy improved wound healing.

### 2.6. Risk of Bias in Individual Studies

The critical appraisal tools recommended by the Joanna Briggs Institute [23] for randomised controlled trials and quasi-experimental studies (non-randomised experimental studies) were used to assess the risk of bias of the included studies. Two reviewers independently evaluated each study, and a third reviewer resolved disagreements. Both tools have the answers ‘yes’, ‘no’, ‘unclear’, or ‘not applicable’ for the questions. For this review, when all answers were ‘yes’, the study was classified as having a low risk of bias, and if any answer was ‘no’ or ‘unclear’, the study was classified as having a risk of bias. No scores were assigned; the results for each question were expressed as the frequency of each classification. In the cases where information was not clear, at least two attempts were made to request additional information from the authors. Evaluation of the risk of bias was not used as a part of the eligibility criteria for article inclusion.

### 2.7. Synthesis of Results

The primary outcome investigated was wound healing after probiotic therapy, and the secondary outcome was the safety of probiotic consumption. To assess the primary outcome, the methods of healing assessments used in each eligible study were examined. The quantitative or qualitative methods used to evaluate the outcome were identified. For the assessment of the secondary outcome, the reported adverse effects in the studies were considered.

## 3. Results

### 3.1. Study Selection

**Figure 1:**
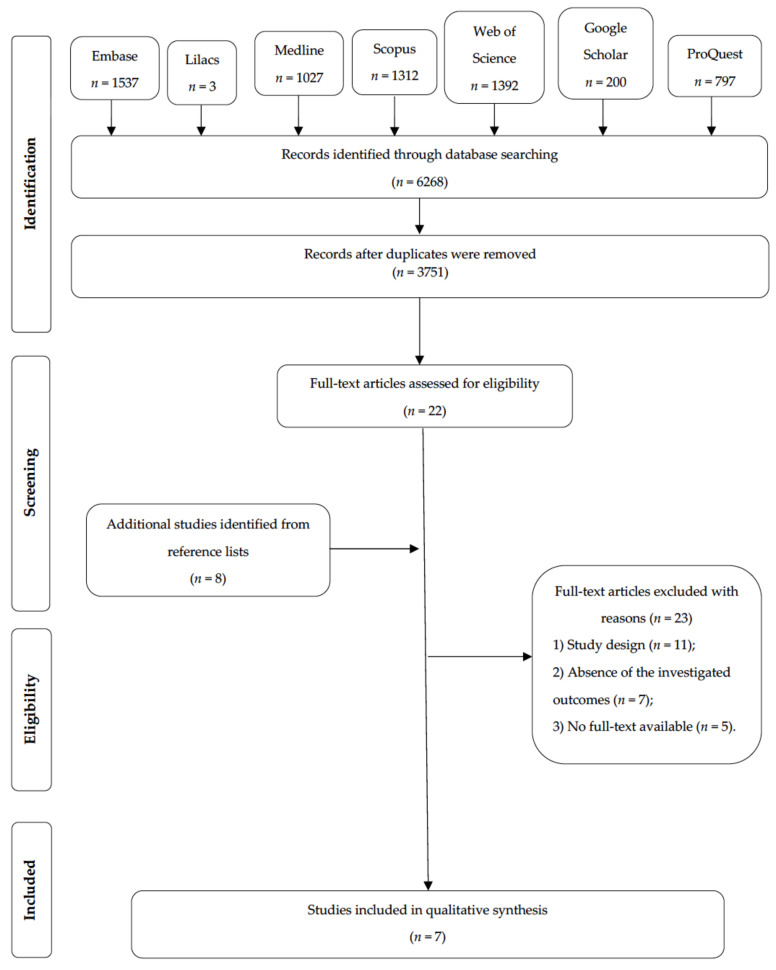
Flow diagram of the literature search and selection criteria.

In the initial search performed using the selected databases, 6268 publications were identified. After the removal of duplicates, the titles and abstracts of 3751 publications were assessed, and 22 studies were selected for full-text reading. From a manual search of the reference lists of the articles, eight more articles were selected for the full-text reading. Finally, seven studies met the eligibility criteria (Figure 1). The reasons for the exclusion of articles are described in Table S2.

### 3.2. Study Characteristics

Table 1 presents the characteristics, objectives, intervention protocols, and main outcomes of the seven studies included in this review [24,25,26,27,28,29,30]. The studies were published between 2014 [24] and 2019 [25], and were carried out in Egypt [26], Italy [27], the United States [28], Iran [29], Pakistan [24], Denmark [30], and Sweden [25]. Three studies assessed the effect of oral/enteral probiotic therapy on burn healing [24,26,28]. El-Ghazely et al., Tahir et al. and Mayes et al. assessed aspects related to skin grafting [24,26,28]. Six studies were randomised clinical trials [25,26,27,28,29,30], and one conducted by Tahir et al. was a placebo-controlled, non-randomised clinical trial [24]. The seven studies included in this review enrolled a total of 348 subjects of both sexes (225 males). The age range was 11 months to 85 years.

Regarding the type of wounds, the studies included burn wounds [24,26,28], surgical wounds [27], diabetic foot ulcers [29], and oral mucosal wounds [25,30]. The intervention protocols were *Lactobacillus reuteri* (DSM 17938 and ATCC PTA 5289) [25,30], *L. fermentum* and *L. delbruekii* [26], *L. rhamnosus* GG (ATCC 53103) [27], *L. rhamnosus* GG [28], *L. acidophilus*, *L. casei*, *L. fermentum* and *Bifidobacterium bifidum* [29], *L. acidophilus* LA-5, *Bifidobacterium* BB-12, *Streptococcus thermophilus* STY-31, and *L. delbrueckii* ssp. *bulgaricus* LBY-27 [24]. The prescription of probiotics in these studies varied with respect to the doses and administration forms, such as sachets [24,26], drops [27], nasoduodenal feeding tube [28], capsules [29], and lozenges [25,30]. The frequency of probiotic therapy in the studies was 1–3 times/day, and the treatment period varied according to the duration of antibiotic use [27], length of hospitalisation [24], the time required to achieve 95% wound healing [28], or the defined research protocol [25,26,29,30].

The healing assessments were conducted locally based either on the need for grafting [26], number of dressing changes per day and post-operative wound complications [27], wound duration and operative days for excision and graft [28], ulcer mean surface area and volume [29], mean body surface area grafted and mean graft loss [24], and healing clinical scores [25,30].

### 3.3. Risk of Bias within Studies

**Figure 2:**
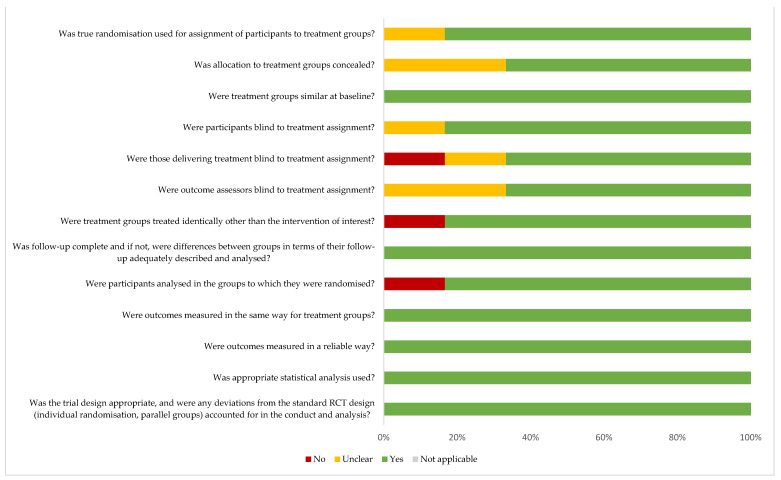
Risk of bias in the included studies (The Joanna Briggs Institute Critical Appraisal Checklist for Randomised Controlled Trials).

Three of the seven studies included in this review [26,29,30] had a low risk of bias. Six parameters of the critical appraisal checklist were met in all randomised controlled clinical trials [25,26,27,28,29,30] (Figure 2). The only non-randomised clinical trial [24] that was included was found to meet seven of the nine parameters assessed using the instrument (Table S3).

### 3.4. Results of Individual Studies

El-Ghazely et al. found that paediatric patients undergoing probiotic therapy had a significantly lower need for grafting; when grafting was not performed, there was a significant decrease in the time required for complete healing of the wound [26]. Tahir et al. found that adult patients receiving probiotic therapy had a larger mean grafted body surface area than those in the control group, but not significantly so. In the same study, the mean graft loss was significantly higher in the control group [24]. In contrast, Mayes et al. did not report differences in the number of operative days for excision and grafting procedures, or the time needed for healing in paediatric patients with or without probiotic therapy [28].

Positive results were also observed for probiotic treatment of surgical wounds [27] and diabetic foot ulcers [29]. Esposito et al. found that the daily frequency of dressing changes was significantly lower in paediatric patients on probiotic therapy (1.7 times/day) than in those in the antibiotics group (3.3 times/day) and in the antibiotics and placebo group (2.8 times/day). In parallel, the incidence of post-operative wound complications was significantly higher in the groups without probiotic therapy [27]. Mohseni et al. observed a significant improvement in the ulcer healing parameters with respect to its length, width, and depth in adults on probiotic therapy [29]. Three studies, two among adults [25,30] and one among paediatric patients [28], did not find improvement in wound healing with probiotic therapy.

It was not possible to conduct a meta-analysis due to the different outcomes assessed across the studies, mainly because of the wide variation in the methods used to evaluate the effects of probiotic therapy, population heterogeneity, type of wounds, and intervention protocols of each study.

## 4. Discussion

The results of this systematic review indicate that there is no consensus on, or high-level evidence for, the effectiveness of probiotic therapy for wound healing owing to the differences in the type of wounds, target population, and criteria for assessing the effect between studies. Nevertheless, it is important to highlight that none of the studies reported adverse effects due to probiotic therapy or a marked increase in the healing time of wounds.

The first studies that dealt with probiotics in dermatology, specifically in atopic dermatitis, were from the first decade of the 2000s. It is therefore an emerging theme that has mainly been researched by means of in vitro and animal studies [31,32,33].

The health-promoting properties of probiotics are suggested to be strain-dependent. The identity and characteristics of the strain are of paramount importance [34], as probiotics may regulate cytokine production and activate antimicrobial immune responses. For instance, some probiotics may induce interleukin (IL)-12, which increases the secretion of interferon (IFN)-γ and activates natural killer (NK) cells. However, they also stimulate the increase of IL-10 that induces antibody production and downregulates the inflammatory response, balancing it and contributing to healing [35]. It is noteworthy that these effects seem to be strain-specific, at least to some degree [36]. *Lactobacillus* strains are capable of inducing pro-inflammatory cytokines such as IL-12 and IFN-γ in addition to anti-inflammatory cytokines such as IL-10 [35], whereas *Bifidobacterium* strains are generally better inducers of IL-10 than *Lactobacillus* strains [37,38]. However, an in vitro study conducted by Dong et al. found little evidence for strain-specific effects of six probiotics on NK cell activity and NK cell or T cell activation. Cytokine production is differentially altered by the probiotic strains of distinct species. Thus, the in vivo biological importance of these strain-specific effects still needs to be elucidated [39].

The studies included in this review that found positive outcomes mainly evaluated the need for grafting, loss of graft, ulcer size, number of dressings per day, and incidence of post-operative wound complications. Meanwhile, when the wounds were assessed using clinical scores, probiotic treatment did not show any significant effect. Skin grafting is the preferred treatment for deep dermal burns, wherein necrotic and inflamed tissues are removed, and faster physiological wound closure is promoted [40]. El-Ghazely et al. found a decrease in the need for grafting in patients treated with probiotics [26]. However, because of the scarcity of studies examining the use of oral probiotics associated with grafting procedures, this effect needs further confirmation. It has already been demonstrated that the strain *L. fermentum* lowers keratinocyte viability and re-epithelialisation in in vitro studies [19,41]. Considering that infection is the second most common cause of graft loss [42], it is possible that therapeutic microorganisms may improve systemic immune functioning [43], favouring the healing process.

The studies included in this review did not elucidate the mechanisms of action of probiotics in the improvement of wound healing. Although the role of the intestinal microbiome in human health and disease is widely known, the role of the skin microbiome in wound healing is less well-defined [44]. Poutahidis et al. identified that oral therapy with probiotics leads to rapid deposition of collagen that is essential for proper wound healing [45]. Yu et al. assessed whether clinical data support the utility of oral and topical probiotics for certain dermatological conditions including chronic wounds. They reported that probiotics can promote the healing process by modulating the inflammatory response and limiting the colonisation of pathogens [16]. A literature review conducted by Lukic et al. identified three possible routes of action of oral probiotic therapy in wound healing. The first pathway is through the central nervous system, where probiotics produce neuroactive molecules and/or modulate the secretory activity of enteroendocrine cells in the intestinal mucosa, leading to the release of neuromodulators with the potential to improve tissue regeneration. The second route is through immunomodulation, in which intestinal probiotics can stimulate the recruitment of lymphocytes to the injured tissue, contributing to the activation of innate and adaptive immune responses. The third route is through the improved absorption of essential nutrients, especially vitamins, minerals, and enzyme cofactors involved in tissue repair to heal skin wounds [8].

Wälivaara et al. [25] and Twetman et al. [30] used the lowest doses of probiotics, in contrast to other studies [24,26,27,28,29], and found no beneficial effects on wound healing. In addition, Wälivaara et al. [25] evaluated the effect of probiotic treatment over a longer period, and Twetman et al. [30] applied a non-validated instrument to assess wound healing. The heterogeneity of these results can also be attributed to differences in the skin microbiome between various regions of the body, such as between drier or more humid areas, or areas with a greater number of sebaceous glands [44]. Beyond this, the studies included in this review did not address the nutritional aspect and gastrointestinal microbiota of individual subjects, although it is known that these factors can influence the wound healing process, as they can affect the immune system. Both malnutrition and dysbiosis are factors that can negatively contribute to proper wound healing [3,4,46]. Therefore, it is important that future studies include the assessment of these factors in wound healing.

Regarding the age range of the patients, previous studies [1,47,48] showed that increasing age is associated with a slight delay in healing, rather than a real loss in its quality. However, it is difficult to identify whether the delay in healing is due to age, or rather diseases that are commonly associated with advancing age. Although the studies included in this review did not evaluate the age of the participants in relation to healing, it is noteworthy that Mohseni et al. found a significant improvement in ulcer healing after probiotic therapy in participants aged 40–85 years [29].

In certain diseases such as epidermolysis bullosa, or in conditions such as diabetic ulcers and extensive burns, in which the healing processes are constant or time-consuming, the need for an effective healing treatment becomes paramount in order to guarantee a better quality of life. Spanos et al. [49] evaluated the impact of the treatment of ulcers and the quality of life of patients with diabetes (*n* = 103) based on the following items: leisure, physical health, dependence/daily life, negative emotions and concerns about ulcers, and discomfort regarding ulcer care. After treatment, the quality of life significantly improved for all assessment items when compared with before treatment. This highlights the importance of effective healing and its relationship to quality of life as an under-researched topic. Therefore, further studies concerning the use of probiotic therapy should be encouraged, since all efforts to favour the healing process can benefit global health and quality of life, which are often compromised in patients with inflammatory skin conditions.

The studies included in this review did not report adverse effects on the clinical risk and safety of oral/enteral probiotic therapy. However, a previous report of more than 600 studies examining the safety of probiotics on microorganisms from six genera, found that, despite the actual probiotic clinical trials showing no evidence of increased risk, the present literature is not well-equipped to answer questions about probiotic safety based on intervention studies with assurance [50].

The strengths of this review are the originality of the study, as well as the methods and expert search strategies used. However, the main limitation is the low certainty of the evidence, due to the small number of studies evaluating the outcomes of interest. This may be because the use of probiotics to treat several types of wounds is an emerging topic of interest. There is also notable variation in the scales used to evaluate the effects of probiotics on wound healing, which made it impossible for us to conduct a meta-analysis.

## 5. Conclusions

We found few studies that have investigated the relationship between probiotic therapy and wound healing. Furthermore, among the studies included in this review, we observed a wide variety of probiotic strains used, types of wounds, and target populations, which prevented us from drawing clear conclusions regarding the effectiveness of probiotic therapy for wound healing. However, we found no marked increase in wound healing time or adverse effects in any of the included studies, highlighting this as a promising field for further clinical investigation.

## Figures and Tables

**Table 1:** Summary of the characteristics and outcomes of the included studies.

Study (Country)	Aim of the Study	Study Design	Subjects/ Patients	Type of Wounds	Intervention Protocol	Healing Assessments	Outcome of Interest
El-Ghazely et al. 2016 (Egypt) [26]	To evaluate the effect of oral probiotic therapy on the outcome of paediatric patients	Prospective, randomised, double-blind, placebo-controlled clinical trial	Total: 40 Group 1 (probiotic): 20 (14 males) Group 2 (control): 20 (12 males) Age: 1–14 years	Thermal burn	**Probiotics**: *Lactobacillus fermentum* and *Lactobacillus delbruekii* **Dosage**: 10 billion colony-forming units (CFU)/sachet **Administration**: sachets **Frequency**: 2 times/day **Placebo group**: starch **Treatment period**: 15 days	Need for grafting	Need for grafting was significantly lower in the probiotic group (probiotics: 10% vs. control: 40%, *p* = 0.028). Significant decrease in the time needed for complete burn wound healing in the probiotic group was observed when the graft was not performed (16.25 ± 0.23 days vs. 20.7 ± 0.51 days, *p* = 0.048).
Esposito et al. 2018 (Italy) [27]	To assess the effectiveness of probiotics as a preventive measure for antibiotic-associated diarrhoea in paediatric patients and its clinical consequences on the post-operative outcome	Prospective, randomised, placebo-controlled trial	Total: 90 (only males) Group 1 (probiotics + antibiotics): 30 Group 2 (antibiotics): 30 Group 3 (antibiotics + placebo): 30 Age: 11–36 months (average 15 months)	Surgical (hypospadias repair)	**Probiotics**: *Lactobacillus rhamnosus* GG (ATCC 53103) **Dosage**: 5 drops of 5 × 10^9^ CFU **Administration**: drops **Frequency**: 1 time/day **Placebo group**: drops of glucose solution at 5% **Treatment period**: approximately 2 h after administration of antibiotics, for 4–16 days, depending on the duration of antibiotic therapy	Number of dressings needed/day and postoperative wound complications	Frequency of dressing change was significantly lower in the probiotic group (average number/day: G1 = 1.7 vs. G2 = 3.3 vs. G3 = 2.8, *p* = 0.001). Incidence of postoperative wound complications was significantly higher in the other groups compared to the probiotic group (G1 = 3.3% vs. G2 = 6.6% vs. G3 = 6.6%, *p* = 0.001).
Mayes et al. 2015 (United States) [28]	To assess the probiotic provision safety in paediatric patients receiving enteral nutrition and to provide a preliminary evaluation of the effect of oral probiotic therapy on clinical outcome	Prospective, randomised, blinded, placebo-controlled trial	Total: 20 Group 1 (probiotic): 10 (6 males) Group 2 (placebo): 10 (8 males) Age: Group 1: 7.1 ± 2.2 years Group 2: 6.9 ± 1.7 years	Burn	**Probiotics**: *Lactobacillus rhamnosus* GG **Dosage**: 15 billion CFU/unit dose **Administration**: nasoduodenal feeding tube **Frequency**: 2 times/day **Placebo group**: identical appearance, with the same inactive ingredient base **Treatment period**: beginning within 10 days of burn and continuing until 95% wound closure was achieved	Wound length of stay (WLOS) and operative days for excision and graft	A reduced healing time was not observed in the probiotic group (probiotic: 0.83 ± 0.1 vs. placebo: 1.02 ± 0.1, *p* < 0.23). There was no difference in the number of operative days for excision and grafting procedures (probiotic: 2.3 ± 0.5 vs. placebo: 3.3 ± 0.6, *p* < 0.23). Clinical safety of oral therapy with probiotics in paediatric patients with burns.
Mohseni et al. 2018 (Iran) [29]	To determine the effects of oral probiotic therapy on wound healing and metabolic status in adult patients with diabetes	Randomised, double-blind, placebo-controlled trial	Total: 60 Group 1 (probiotic): 30 (20 males) Group 2 (placebo): 30 (20 males) Age: 40–85 years	Diabetic foot ulcer	**Probiotics**: *Lactobacillus acidophilus, Lactobacillus casei, Lactobacillus fermentum,* and *Bifidobacterium bifidum* **Dosage**: 2 × 10^9^ CFU/g **Administration**: capsule **Frequency**: daily **Placebo group**: not informed **Treatment period**: 12 weeks	Mean ulcer area and ulcer volume	Significant improvement in parameters of wound healing in the probiotic group (ulcer length: −1.3 ± 0.9 vs. −0.8 ± 0.7 cm, *p* = 0.01; width: −1.1 ± 0.7 vs. −0.7 ± 0.7 cm, *p* = 0.02; depth: −0.5 ± 0.3 vs. −0.3 ± 0.3 cm, *p* = 0.02).
Tahir et al. 2014 (Pakistan) [24]	To find an alternate, effective method to reduce infection, predict graft take, and minimise hospital stay in adult patients	Prospective, placebo-controlled trial, not randomised	Total: 64 Group 1 (probiotic): 22 (10 males) Group 2 (control): 42 (12 males) Age: Group 1: 28.2 ± 10.2 years Group 2: 30.2 ± 13.80 years	Burn	**Probiotics**: *Lactobacillus acidophilus* LA-5, *Bifidobacterium* BB-12, *Streptococcus thermophilus* STY-31, and *Lactobacillus delbrueckii* ssp. *bulgaricus* LBY-27 **Dosage**: 2 g of >8 billion CFU/sachet **Administration**: sachets **Frequency**: 2 times/day **Control group**: nothing **Treatment period**: started on day 2 of admission and continued during the entire period of hospitalisation	Mean body surface area grafted and mean graft loss	Mean body surface area grafted for each patient was higher in the probiotic group (probiotics: 10.81% vs. control: 9.75%, *p* = 0.0917). Mean graft loss was higher in the control group (probiotics: 20.14% vs. control: 29.26%, *p* = 0.0093).
Twetman et al. 2018 (Denmark) [30]	To investigate the impact of topical and systemic applications of probiotic lactobacilli on the healing of standardised wounds in adult patients	Randomised, placebo-controlled, double-blind, cross-over design	Total: 10 (2 males) Age: Mean age: 29.5 years (range 21–66 years)	Oral mucosa	**Probiotics**: *Lactobacillus reuteri* (DSM 17938 and ATCC PTA 5289) **Dosage**: at least 5 × 10^8^ live bacteria of each strain/lozenge **Administration**: lozenges **Frequency**: 2 times/day **Placebo group**: lozenges had an identical composition, shape, and taste, but without active bacteria **Treatment period**: 8 days before the biopsy and a further 8 days after	Four-level clinical score	No statistically significant differences in the oral wound healing pattern between test and placebo. *
Wälivaara et al. 2019 (Sweden) [25]	To investigate the effect of oral probiotic therapy on oral wound healing and to assess local bacterial growth and the postoperative concentrations of oxytocin in saliva	Randomised placebo-controlled trial	Total: 64 (31 males) Group 1 (probiotic): 30 Group 2 (placebo): 31 Age: Mean age: 29.9 years (range 18–34)	Oral mucosa	**Probiotics**: *Lactobacillus reuteri* (DSM 17938 and ATCC PTA 5289) **Dosage**: at least 2 × 10^8^ live bacteria **Administration**: lozenges **Frequency**: 3 times/day (one in the morning, one at lunchtime, and one in the evening) **Placebo group**: lozenges had an identical colour, shape, and taste, but without active bacteria **Treatment period**: 2 weeks	Clinical healing index scores	No differences between the groups in the distribution of the healing scores.*

CFU: colony-forming units, WLOS: was defined as the point in recovery when the wounds were 95% closed, * does not report a *p*-value.

## Supplementary Materials

The following are available online at https://www.mdpi.com/article/10.3390/nu14010111/s1, Table S1: Database search strategy; Table S2: Full-text articles excluded with reasons (*n* = 23); and Table S3: Risk of bias in the included studies—Critical Appraisal Checklist for Randomised Controlled Trials and for Quasi-Experimental Studies (non-randomised experimental studies).
