# Supplementary material for: Does Probiotic Consumption Enhance Wound Healing? A Systematic Review
Source: Nutrients. 2021 Dec 27;14(1):111. doi: 10.3390/nu14010111 (PMC8746682; doi:10.3390/nu14010111)
Supplement: Supplementary file 1 [file nutrients-14-00111-s001.zip › Table S1.pdf]

Table S1. Database search strategy.

DATABASE	SEARCH (November 30, 2020)
MEDLINE	((("Cutaneous wounds"[All Fields] OR "Thermal burn"[All Fields] OR "Burn injury"[All Fields] OR ("burns"[MeSH Terms] OR "burns"[All Fields]) OR ("injuries"[Subheading] OR "injuries"[All Fields] OR "wounds"[All Fields] OR "wounds and injuries"[MeSH Terms] OR ("wounds"[All Fields] AND "injuries"[All Fields]) OR "wounds and injuries"[All Fields]) OR "Skin wounds"[All Fields] OR "Wound healing"[All Fields] OR ("cicatrix"[MeSH Terms] OR "cicatrix"[All Fields]) OR "Granulation Tissue"[All Fields] OR ("regeneration"[MeSH Terms] OR "regeneration"[All Fields]) OR "Re-epithelialization"[All Fields] OR ("keratinocytes"[MeSH Terms] OR "keratinocytes"[All Fields] OR "keratinocyte"[All Fields]) OR ("fibroblasts"[MeSH Terms] OR "fibroblasts"[All Fields])) AND (("prebiotics"[MeSH Terms] OR "prebiotics"[All Fields]) OR ("probiotics"[MeSH Terms] OR "probiotics"[All Fields]) OR ("probiotics"[MeSH Terms] OR "probiotics"[All Fields] OR "probiotic"[All Fields]) OR ("prebiotics"[MeSH Terms] OR "prebiotics"[All Fields] OR "prebiotic"[All Fields]) OR ("synbiotics"[MeSH Terms] OR "synbiotics"[All Fields] OR "synbiotic"[All Fields]) OR ("synbiotics"[MeSH Terms] OR "synbiotics"[All Fields]) OR Symbiotic[All Fields] OR Symbiotics[All Fields] OR Bioproducts[All Fields] OR ("yogurt"[MeSH Terms] OR "yogurt"[All Fields]) OR ("yogurt"[MeSH Terms] OR "yogurt"[All Fields] OR "yoghurt"[All Fields]) OR "Lactic acid bacteria"[All Fields] OR "Streptococcus thermophilus"[All Fields] OR "S. thermophilus"[All Fields] OR "Fermented milk"[All Fields] OR ("bifidobacterium"[MeSH Terms] OR "bifidobacterium"[All Fields]) OR ("lactobacillus"[MeSH Terms] OR "lactobacillus"[All Fields]) OR ("lactococcus"[MeSH Terms] OR "lactococcus"[All Fields]) OR ("saccharomyces"[MeSH Terms] OR "saccharomyces"[All Fields]) OR "Bacillus mesentericus"[All Fields] OR "B. mesentericus"[All Fields] OR "Prebiotics supplementation"[All Fields] OR "Probiotics supplementation"[All Fields] OR "Probiotic supplementation"[All Fields] OR "Prebiotic supplementation"[All Fields] OR "Synbiotic supplementation"[All Fields] OR "Synbiotics supplementation"[All Fields] OR "Symbiotic supplementation"[All Fields] OR (Symbiotics[All Fields] AND supplementation[All Fields]) OR (Bioproducts[All Fields] AND supplementation[All Fields]) OR "Yogurt supplementation"[All Fields] OR "yoghurt supplementation"[All Fields] OR "lactic acid bacteria supplementation"[All Fields] OR (("streptococcus thermophilus"[MeSH Terms] OR ("streptococcus"[All Fields] AND "thermophilus"[All Fields]) OR "streptococcus thermophilus"[All Fields]) AND supplementation[All Fields]) OR (S.[All Fields] AND thermophilus[All Fields] AND supplementation[All Fields]) OR "fermented milk supplementation"[All Fields] OR (("bifidobacterium"[MeSH Terms] OR "bifidobacterium"[All Fields]) AND supplementation[All Fields]) OR "Lactobacillus supplementation"[All Fields] OR (("lactococcus"[MeSH Terms] OR "lactococcus"[All Fields]) AND supplementation[All Fields]) OR (("saccharomyces"[MeSH Terms] OR "saccharomyces"[All Fields]) AND supplementation[All Fields]) OR (("bacillus pumilus"[MeSH Terms] OR ("bacillus"[All Fields] AND "pumilus"[All Fields]) OR "bacillus pumilus"[All Fields] OR ("bacillus"[All Fields] AND "mesentericus"[All Fields]) OR "bacillus mesentericus"[All Fields]) AND supplementation[All Fields]) OR (B.[All Fields] AND mesentericus[All Fields] AND supplementation[All Fields]) OR (("prebiotics"[MeSH Terms] OR "prebiotics"[All Fields]) AND intake[All Fields]) OR "Probiotics intake"[All Fields] OR "Probiotic intake"[All Fields] OR "Prebiotic intake"[All Fields] OR "Synbiotic intake"[All Fields] OR (("synbiotics"[MeSH

	Terms] OR "synbiotics"[All Fields]) AND intake[All Fields]) OR (Symbiotic[All Fields] AND intake[All Fields]) OR (Symbiotics[All Fields] AND intake[All Fields]) OR (bioproducts[All Fields] AND intake[All Fields]) OR "yogurt intake"[All Fields] OR "yoghurt intake"[All Fields] OR (("lactobacillales"[MeSH Terms] OR "lactobacillales"[All Fields] OR ("lactic"[All Fields] AND "acid"[All Fields] AND "bacteria"[All Fields]) OR "lactic acid bacteria"[All Fields]) AND intake[All Fields]) OR (("streptococcus thermophilus"[MeSH Terms] OR ("streptococcus"[All Fields] AND "thermophilus"[All Fields]) OR "streptococcus thermophilus"[All Fields]) AND intake[All Fields]) OR (S.[All Fields] AND thermophilus[All Fields] AND intake[All Fields]) OR "fermented milk intake"[All Fields] OR (("bifidobacterium"[MeSH Terms] OR "bifidobacterium"[All Fields]) AND intake[All Fields]) OR (("lactobacillus"[MeSH Terms] OR "lactobacillus"[All Fields]) AND intake[All Fields]) OR (("lactococcus"[MeSH Terms] OR "lactococcus"[All Fields]) AND intake[All Fields]) OR (("saccharomyces"[MeSH Terms] OR "saccharomyces"[All Fields]) AND intake[All Fields]) OR (("bacillus pumilus"[MeSH Terms] OR ("bacillus"[All Fields] AND "pumilus"[All Fields]) OR "bacillus pumilus"[All Fields] OR ("bacillus"[All Fields] AND "mesentericus"[All Fields]) OR "bacillus mesentericus"[All Fields]) AND intake[All Fields]) OR (B.[All Fields] AND mesentericus[All Fields] AND intake[All Fields]) OR (("prebiotics"[MeSH Terms] OR "prebiotics"[All Fields]) AND ("organization and administration"[MeSH Terms] OR ("organization"[All Fields] AND "administration"[All Fields]) OR "organization and administration"[All Fields] OR "administration"[All Fields])) OR "Probiotics administration"[All Fields] OR "Probiotic administration"[All Fields] OR "Prebiotic administration"[All Fields] OR "Synbiotic administration"[All Fields] OR "Synbiotics administration"[All Fields] OR "Symbiotic administration"[All Fields] OR (Symbiotics[All Fields] AND ("organization and administration"[MeSH Terms] OR ("organization"[All Fields] AND "administration"[All Fields]) OR "organization and administration"[All Fields] OR "administration"[All Fields])) OR (Bioproducts[All Fields] AND ("organization and administration"[MeSH Terms] OR ("organization"[All Fields] AND "administration"[All Fields]) OR "organization and administration"[All Fields] OR "administration"[All Fields])) OR (("yogurt"[MeSH Terms] OR "yogurt"[All Fields]) AND ("organization and administration"[MeSH Terms] OR ("organization"[All Fields] AND "administration"[All Fields]) OR "organization and administration"[All Fields] OR "administration"[All Fields])) OR "Yoghurt administration"[All Fields] OR (("lactobacillales"[MeSH Terms] OR "lactobacillales"[All Fields] OR ("lactic"[All Fields] AND "acid"[All Fields] AND "bacteria"[All Fields]) OR "lactic acid bacteria"[All Fields]) AND ("organization and administration"[MeSH Terms] OR ("organization"[All Fields] AND "administration"[All Fields]) OR "organization and administration"[All Fields] OR "administration"[All Fields])) OR (("streptococcus thermophilus"[MeSH Terms] OR ("streptococcus"[All Fields] AND "thermophilus"[All Fields]) OR "streptococcus thermophilus"[All Fields]) AND ("organization and administration"[MeSH Terms] OR ("organization"[All Fields] AND "administration"[All Fields]) OR "organization and administration"[All Fields] OR "administration"[All Fields])) OR (S.[All Fields] AND thermophilus[All Fields] AND ("organization and administration"[MeSH Terms] OR ("organization"[All Fields] AND "administration"[All Fields]) OR "organization and administration"[All Fields] OR "administration"[All Fields])) OR "Fermented milk administration"[All Fields] OR (("bifidobacterium"[MeSH Terms] OR "bifidobacterium"[All Fields]) AND ("organization and administration"[MeSH Terms] OR ("organization"[All Fields] AND "administration"[All Fields]) OR "organization and administration"[All Fields] OR "administration"[All Fields])) OR "Lactobacillus administration "[All Fields] OR

	(("lactococcus"[MeSH Terms] OR "lactococcus"[All Fields]) AND ("organization and administration"[MeSH Terms] OR ("organization"[All Fields] AND "administration"[All Fields]) OR "organization and administration"[All Fields] OR "administration"[All Fields])) OR (("saccharomyces"[MeSH Terms] OR "saccharomyces"[All Fields]) AND ("organization and administration"[MeSH Terms] OR ("organization"[All Fields] AND "administration"[All Fields]) OR "organization and administration"[All Fields] OR "administration"[All Fields])) OR (("bacillus pumilus"[MeSH Terms] OR ("bacillus"[All Fields] AND "pumilus"[All Fields]) OR "bacillus pumilus"[All Fields] OR ("bacillus"[All Fields] AND "mesentericus"[All Fields]) OR "bacillus mesentericus"[All Fields]) AND ("organization and administration"[MeSH Terms] OR ("organization"[All Fields] AND "administration"[All Fields]) OR "organization and administration"[All Fields] OR "administration"[All Fields])) OR (B.[All Fields] AND mesentericus[All Fields] AND ("organization and administration"[MeSH Terms] OR ("organization"[All Fields] AND "administration"[All Fields]) OR "organization and administration"[All Fields] OR "administration"[All Fields])))) AND (("humans"[MeSH Terms] OR "humans"[All Fields] OR "human"[All Fields]) OR ("adult"[MeSH Terms] OR "adult"[All Fields] OR "adults"[All Fields]) OR ("adult"[MeSH Terms] OR "adult"[All Fields]) OR ("child"[MeSH Terms] OR "child"[All Fields]) OR ("child"[MeSH Terms] OR "child"[All Fields] OR "children"[All Fields]) OR ("aged"[MeSH Terms] OR "aged"[All Fields] OR "elderly"[All Fields]) OR ("women"[MeSH Terms] OR "women"[All Fields]) OR ("women"[MeSH Terms] OR "women"[All Fields] OR "woman"[All Fields]) OR ("men"[MeSH Terms] OR "men"[All Fields]) OR ("men"[MeSH Terms] OR "men"[All Fields] OR "man"[All Fields]) OR ("male"[MeSH Terms] OR "male"[All Fields]) OR ("female"[MeSH Terms] OR "female"[All Fields]))) NOT ("animals"[MeSH Terms:noexp] OR animals[All Fields])
**EMBASE**	('cutaneous wounds' OR 'thermal burn' OR 'burn injury' OR burns OR wounds OR 'skin wounds' OR 'wound healing' OR cicatrix OR 'granulation tissue' OR regeneration OR 're-epithelialization' OR keratinocyte OR fibroblasts) AND (prebiotics OR probiotics OR probiotic OR prebiotic OR synbiotic OR synbiotics OR symbiotic OR symbiotics OR bioproducts OR yogurt OR yoghurt OR 'lactic acid bacteria' OR 'streptococcus thermophilus' OR 's. thermophilus' OR 'fermented milk' OR bifidobacterium OR lactobacillus OR lactococcus OR saccharomyces OR 'bacillus mesentericus' OR 'b. mesentericus' OR 'prebiotics supplementation' OR 'probiotics supplementation' OR 'probiotic supplementation' OR 'prebiotic supplementation' OR 'synbiotic supplementation' OR 'synbiotics supplementation' OR 'symbiotic supplementation' OR 'symbiotics supplementation' OR 'bioproducts supplementation' OR 'yogurt supplementation' OR 'yoghurt supplementation' OR 'lactic acid bacteria supplementation' OR 'streptococcus thermophilus supplementation' OR 's. thermophilus supplementation' OR 'fermented milk supplementation' OR 'bifidobacterium supplementation' OR 'lactobacillus supplementation' OR 'lactococcus supplementation' OR 'saccharomyces supplementation' OR 'bacillus mesentericus supplementation' OR 'b. mesentericus supplementation' OR 'prebiotics intake' OR 'probiotics intake' OR 'probiotic intake' OR 'prebiotic intake' OR 'synbiotic intake' OR 'synbiotics intake' OR 'symbiotic intake' OR 'symbiotics intake' OR 'bioproducts intake' OR 'yogurt intake' OR 'yoghurt intake' OR 'lactic acid bacteria intake' OR 'streptococcus thermophilus intake' OR 's. thermophilus intake' OR 'fermented milk intake' OR 'bifidobacterium intake' OR 'lactobacillus intake' OR 'lactococcus intake' OR 'saccharomyces intake' OR 'bacillus mesentericus intake' OR 'b. mesentericus intake' OR 'prebiotics administration' OR 'probiotics administration' OR 'probiotic administration' OR

	'prebiotic administration' OR 'synbiotic administration' OR 'synbiotics administration' OR 'symbiotic administration' OR 'symbiotics administration' OR 'bioproducts administration' OR 'yogurt administration' OR 'yoghurt administration' OR 'lactic acid bacteria administration' OR 'streptococcus thermophilus administration' OR 's. thermophilus administration' OR 'fermented milk administration' OR 'bifidobacterium administration' OR 'lactobacillus administration' OR 'lactococcus administration' OR 'saccharomyces administration' OR 'bacillus mesentericus administration' OR 'b. mesentericus administration') AND (human OR adults OR adult OR child OR children OR elderly OR women OR woman OR men OR man OR male OR female) NOT animals:af
**LILACS**	tw:((tw:((tw:(feridas OR cicatrização)))) AND (tw:((tw:(prebióticos OR probióticos OR simbióticos))))) AND (db:("LILACS"))
**SCOPUS**	(TITLE-ABS-KEY ("Cutaneous wounds" OR "Thermal burn" OR "Burn injury" OR burns OR wounds OR "Skin wounds" OR "Wound healing" OR cicatrix OR "Granulation Tissue" OR regeneration OR "Re-epithelialization" OR keratinocyte OR fibroblasts) AND TITLE-ABS-KEY (prebiotics OR probiotics OR synbiotics OR symbiotics OR bioproducts OR yogurt OR "Lactic acid bacteria" OR "Streptococcus thermophilus" OR "Fermented milk" OR bifidobacterium OR lactobacillus OR lactococcus OR saccharomyces OR "Bacillus mesentericus") AND TITLE-ABS-KEY (human OR adults OR adult OR child OR children OR elderly OR women OR woman OR men OR man OR male OR female) AND NOT TITLE-ABS-KEY (animals))
**WEB OF SCIENCE**	TS = ("Cutaneous wounds" OR "Thermal burn" OR "Burn injury" OR Burns OR Wounds OR "Skin wounds" OR "Wound healing" OR Cicatrix OR "Granulation Tissue" OR Regeneration OR "Re-epithelialization" OR Keratinocyte OR Fibroblasts) AND TS = (Prebiotics OR Probiotics OR Probiotic OR Prebiotic OR Synbiotic OR Synbiotics OR Symbiotic OR Symbiotics OR Bioproducts OR Yogurt OR Yoghurt OR "Lactic acid bacteria" OR "Streptococcus thermophilus" OR "S. thermophilus" OR "Fermented milk" OR Bifidobacterium OR Lactobacillus OR Lactococcus OR Saccharomyces OR "Bacillus mesentericus" OR "B. mesentericus" OR "Prebiotics supplementation" OR "Probiotics supplementation" OR "Probiotic supplementation" OR "Prebiotic supplementation" OR "Synbiotic supplementation" OR "Synbiotics supplementation" OR "Symbiotic supplementation" OR "Symbiotics supplementation" OR "Bioproducts supplementation" OR "Yogurt supplementation" OR "yoghurt supplementation" OR "lactic acid bacteria supplementation" OR "Streptococcus thermophilus supplementation" OR "S. thermophilus supplementation" OR "fermented milk supplementation" OR " Bifidobacterium supplementation" OR "Lactobacillus supplementation" OR "Lactococcus supplementation" OR "Saccharomyces supplementation" OR "Bacillus mesentericus supplementation" OR "B. mesentericus supplementation" OR "Prebiotics intake" OR "Probiotics intake" OR "Probiotic intake" OR "Prebiotic intake" OR "Synbiotic intake" OR "Synbiotics intake" OR "Symbiotic intake" OR "Symbiotics intake" OR "bioproducts intake" OR "yogurt intake" OR "yoghurt intake" OR "lactic acid bacteria intake" OR "Streptococcus thermophilus intake" OR "S. thermophilus intake" OR "fermented milk intake" OR "Bifidobacterium intake" OR "Lactobacillus intake" OR "Lactococcus intake" OR "Saccharomyces intake" OR "Bacillus mesentericus intake" OR "B. mesentericus intake" OR "Prebiotics administration" OR "Probiotics administration" OR "Probiotic administration" OR "Prebiotic administration" OR "Synbiotic administration" OR "Synbiotics administration" OR "Symbiotic

	administration" OR "Symbiotics administration" OR "Bioproducts administration" OR "Yogurt administration" OR "Yoghurt administration" OR "Lactic acid bacteria administration" OR "Streptococcus thermophilus administration" OR "S. thermophilus administration" OR "Fermented milk administration" OR "Bifidobacterium administration" OR "Lactobacillus administration "OR "Lactococcus administration" OR "Saccharomyces administration" OR "Bacillus mesentericus administration" OR "B. mesentericus administration") AND TS = (Human OR Adults OR Adult OR Child OR Children OR Elderly OR Women OR Woman OR Men OR Man OR Male OR Female) NOT TS=(Animals)
**CLINICAL TRIALS**	prebiotics OR probiotics OR probiotic OR prebiotic OR synbiotic OR synbiotics OR symbiotic OR symbiotics OR bioproducts OR yogurt OR yoghurt OR 'lactic acid bacteria' OR 'streptoccus thermophilus' OR 's. thermophilus' OR 'fermented milk' | Completed, Terminated Studies | ('cutaneous wounds' OR 'thermal burn' OR 'burn injury' OR burns OR wounds OR 'skin wounds' OR 'wound healing' OR cicatrix OR 'granulation tissue' OR regeneration OR 're-epithelialization' OR keratinocyte OR fibroblasts)
**GOOGLE SCHOLAR**	With all of the words: Healing<br>With at least one of the words: Prebiotics Probiotics Synbiotics Symbiotics Bioproducts Yogurt "Lactic acid bacteria" "Streptococcus thermophilus" "Fermented milk" Bifidobacterium<br>Without the words: Animals<br>Where my words occurs: anywhere in the article<br>200 most relevant hits
**ProQuest**	ab("Cutaneous wounds" OR "Thermal burn" OR "Burn injury" OR Burns OR Wounds OR "Skin wounds" OR "Wound healing" OR Cicatrix OR "Granulation Tissue" OR Regeneration OR "Re-epithelialization" OR Keratinocyte OR Fibroblasts) AND ab(Prebiotics OR Probiotics OR Probiotic OR Prebiotic OR Synbiotic OR Synbiotics OR Symbiotic OR Symbiotics OR Bioproducts OR Yogurt OR Yoghurt OR "Lactic acid bacteria" OR "Streptococcus thermophilus" OR "S. thermophilus" OR "Fermented milk" OR Bifidobacterium OR Lactobacillus OR Lactococcus OR Saccharomyces OR "Bacillus mesentericus" OR "B. mesentericus" OR "Prebiotics supplementation" OR "Probiotics supplementation" OR "Probiotic supplementation" OR "Prebiotic supplementation" OR "Synbiotic supplementation" OR "Synbiotics supplementation" OR "Symbiotic supplementation" OR "Symbiotics supplementation" OR "Bioproducts supplementation" OR "Yogurt supplementation" OR "yoghurt supplementation" OR "lactic acid bacteria supplementation" OR "Streptococcus thermophilus supplementation" OR "S. thermophilus supplementation" OR "fermented milk supplementation" OR " Bifidobacterium supplementation" OR "Lactobacillus supplementation" OR "Lactococcus supplementation" OR "Saccharomyces supplementation" OR "Bacillus mesentericus supplementation" OR "B. mesentericus supplementation" OR "Prebiotics intake" OR "Probiotics intake" OR "Probiotic intake" OR "Prebiotic intake" OR "Synbiotic intake" OR "Synbiotics intake" OR "Symbiotic intake" OR "Symbiotics intake" OR "bioproducts intake" OR "yogurt intake" OR "yoghurt intake" OR "lactic acid bacteria intake" OR "Streptococcus thermophilus intake" OR "S. thermophilus intake" OR "fermented milk intake" OR "Bifidobacterium intake" OR "Lactobacillus intake" OR "Lactococcus intake" OR "Saccharomyces intake" OR "Bacillus mesentericus intake" OR "B. mesentericus intake" OR "Prebiotics administration" OR "Probiotics administration" OR "Probiotic administration" OR "Prebiotic administration" OR "Synbiotic administration" OR "Synbiotics administration" OR "Symbiotic administration" OR "Symbiotics administration" OR "Bioproducts administration" OR "Yogurt administration" OR

	"Yoghurt administration" OR "Lactic acid bacteria administration" OR "Streptococcus thermophilus administration" OR "S. thermophilus administration" OR "Fermented milk administration" OR "Bifidobacterium administration" OR "Lactobacillus administration "OR "Lactococcus administration" OR "Saccharomyces administration" OR "Bacillus mesentericus administration" OR "B. mesentericus administration") AND (Human OR Adults OR Adult OR Child OR Children OR Elderly OR Women OR Woman OR Men OR Man OR Male OR Female) NOT animals
