# Supplementary material for: Does Probiotic Consumption Enhance Wound Healing? A Systematic Review
Source: Nutrients. 2021 Dec 27;14(1):111. doi: 10.3390/nu14010111 (PMC8746682; doi:10.3390/nu14010111)
Supplement: Supplementary file 1 [file nutrients-14-00111-s001.zip › Table S2.pdf]

Table S2. Full-text articles excluded with reasons (n = 23).

Author, Year	Reason for exclusion
Aggour, 2020(1); Aswath, 2014(2); Kashef, 2020(6); Mueller, 2019(11); Narang, 2016(12); Nirmala, 2019(15); Pedersen, 2019(16); Ramos, 2010(18); Ramos, 2012(19); Tasli, 2006(21); Trinchieri, 2011(23)	Study design
Elsadek, 2020(3); Ghalambor, 2009(4); Koren, 2007(8); Kotzampassi, 2006(9); Navarro-López, 2018(14); Pudgar, 2020(17); Thomson, 2012(22)	Absence of the investigated outcomes
Jacques, 1966(5); Klimenko, 2002(7); Lazareva, 2002(10); Nasrabadi, 2012(13); Shul'gin, 2007(20)	No full-text available

REFERENCES

1. Aggour RL, Mahmoud SH, Abdelwhab A. Evaluation of the effect of probiotic lozenges in the treatment of recurrent aphthous stomatitis: a randomized, controlled clinical trial. *Clinical Oral Investigations* 2020.

2. Aswath N, Praveen KST, Raghavendra JS *et al*. A randomized, open label, clinical study of synbiotics in patients with recurrent minor aphthous ulcers. *Research Journal of Pharmaceutical, Biological and Chemical Sciences* 2014; 5(2):1900-1905.

3. Elsadek MF, Ahmed BM, Alkhawtani DM *et al*. A comparative clinical, microbiological and glycemic analysis of photodynamic therapy and *Lactobacillus reuteri* in the treatment of chronic periodontitis in type-2 diabetes mellitus patients. *Photodiagnosis and Photodynamic Therapy* 2020.

4. Ghalambor A, Pipelzadeh MH. Clinical study on the efficacy of orally administered crushed fresh garlic in controlling *Pseudomonas aeruginosa* infection in burn patients with varying burn degrees. *Jundishapur Journal of Microbiology* 2009; 2(1): 7-13.

5. Jacques L. Action of lactic bacilli on the ph, infection, and the cicatrization of skin wounds in the rat and man. *Semaine thérapeutique* 1966; 42(3):163-169.

6. Kashef N. Probiotics in skin wound healing. *Razi Journal of Medical Sciences* 2020; 26(12):43-55.

7. Klimenko VN, Tugushev AS, Zakharchuk AV *et al.* Application of probiotics in the treatment of patients with nonhealing purulent-inflammatory wounds. *Klinichna khirurhiia / Ministerstvo okhorony zdorov'ia Ukraïny, Naukove tovarystvo khirurhiv Ukraïny* 2002; 0(11):33-34.

8. Koren L, Gurfinkel R, Glezinger R *et al.* The effect of *Lactobacillus* bacteria supplement on sepsis and its complications in patients with acute burns. *Burns* 2007; 33(5):594-598.

9. Kotzampassi K, Giamarellos-Bourboulis EJ, Voudouris A *et al.* Benefits of a Synbiotic Formula (Synbiotic 2000Forte®) in Critically Ill Trauma Patients: Early Results of a Randomized Controlled Trial. *World Journal of Surgery* 2006; 30(10):1848-55.

10. Lazareva EB, Smirnov SV, Khvatov VB *et al.* [Oral administration of pectins for prophylaxis and treatment of purulent septic complications in patients with burns]. Tt - peroral'noe primenenie pektinov dlia profilaktiki i lecheniia gnoĭno-septicheskikh oslozhneniĭ u ozhogovykh bol'nykh. *Antibiotiki i khimioterapiia = Antibiotics and chemoterapy* 2002; 47(4):16-19.

11. Mueller SA, Mayer C, Bojaxhiu B *et al*. Effect of preoperative immunonutrition on complications after salvage surgery in head and neck cancer. *Journal of Otolaryngology – Head & Neck Surgery* 2019; 48:25.

12. Narang R, Hull T, Perrins S *et al.* Should Immunomodulation Therapy Alter the Surgical Management in Patients With Rectovaginal Fistula and Crohn's Disease?. *Diseases of the Colon and Rectum* 2016; 59(7):670-676.

13. Nasrabadi MH, Zahedi F. Effect of Probiotics on Cutaneous Wound Healing. National Congress of Probiotic and Functional Foods; 2012; Iran.

14. Navarro-López V, Ramírez-Boscá A, Ramón-Vidal D *et al.* Effect of Oral Administration of a Mixture of Probiotic Strains on SCORAD Index and Use of Topical Steroids in Young Patients With Moderate Atopic Dermatitis A Randomized Clinical Trial. *JAMA Dermatology* 2018; 154(1):37-43.

15. Nirmala M, Smitha SG, Kamath GJ. A Study to Assess The Efficacy of Local Application of Oral Probiotic in Treating Recurrent Aphthous Ulcer and Oral Candidiasis. *Indian Journal of Otolaringology and Head & Neck Surgery* 2019; 71(Suppl 1): S113-S117.

16. Pedersen AML, Bukkehave KH, Bennett EP *et al*. Effect of Lozenges Containing *Lactobacillus reuteri* on the Severity of Recurrent Aphthous Ulcers: a Pilot Study. *Probiotics and Antimicrobial Proteins* 2020; 12(3):819-823.

17. Pudgar P, Povšič K, Čuk K *et al*. Probiotic strains of *Lactobacillus brevis* and *Lactobacillus plantarum* as adjunct to non-surgical periodontal therapy: 3-month results of a randomized controlled clinical trial. *Clinical Oral Investigation* 2020.

18. Ramos AN, Gobbato N, Rachid M *et al.* Effect of *Lactobacillus plantarum* and *Pseudomonas aeruginosa* culture supernatants on polymorphonuclear damage and inflammatory response. *International Immunopharmacology* 2010; 10(2):247-251.

19. Ramos AN, Sesto Cabral ME, Noseda D *et al.* Antipathogenic properties of *Lactobacillus plantarum* on *Pseudomonas aeruginosa*: The potential use of its supernatants in the treatment of infected chronic wounds. *Wound Repair and Regeneration* 2012; 20(4):552-562.

20. Shul'gin Iu P, Koval' PV, Usov VV *et al.* [Application of cultured milk products with lavinariia additives to decrease iodine deficit for surgical patients]. *Voprosy pitaniia* 2007; 76(1):67-69.

21. Tasli L, Mat C, De Simone C *et al*. *Lactobacilli* lozenges in the management of oral ulcers of Behçet's syndrome. *Clinical and Experimental Rheumatology* 2006; 24 (Suppl. 42):S83-S86.

22. Thomson CH, Hassan I, Dunn K. Yakult: a role in combating multi-drug resistant *Pseudomonas aeruginosa*?. *Journal of Wound Care* 2012; 21(11):566-569.

23. Trinchieri V, Di Carlo S, Bossu' M *et al*. Use of Lozenges Containing *Lactobacillus brevis* CD2 in Recurrent Aphthous Stomatitis: A Double-Blind Placebo-Controlled Trial. *Ulcers* 2011.
