# Supplementary material for: Does Probiotic Consumption Enhance Wound Healing? A Systematic Review
Source: Nutrients. 2021 Dec 27;14(1):111. doi: 10.3390/nu14010111 (PMC8746682; doi:10.3390/nu14010111)
Supplement: Supplementary file 1 [file nutrients-14-00111-s001.zip › Table S3.pdf]

Table S3. Risk of bias in the included studies - Critical Appraisal Checklist for Randomised Controlled Trials and for Quasi-Experimental Studies (non-randomised experimental studies).

	Article Author/Year						
**Questions**	**El-Ghazely et al., 2016**	**Esposito et al., 2018**	**Mayes et al., 2015**	**Mohseni et al., 2018**	**Twetman et al., 2018**	**Wälivaara et al., 2019**	**Tahir et al., 2014***
1. Was true randomisation used for assignment of participants to treatment groups?	Y	U	Y	Y	Y	Y	-
2. Was allocation to treatment groups concealed?	Y	U	U	Y	Y	Y	-
3. Were treatment groups similar at baseline?/Were the participants included in any comparisons similar?*	Y	Y	Y	Y	Y	Y	Y
4. Were participants blind to treatment assignment?	Y	U	Y	Y	Y	Y	-
5. Were those delivering treatment blind to treatment assignment?	Y	N	U	Y	Y	Y	-
6. Were outcome assessors blind to treatment assignment?	Y	U	U	Y	Y	Y	-
7. Were treatment groups treated identically other than the intervention of interest?/Were the participants included in any comparisons receiving similar treatment/care, other than the	Y	N	Y	Y	Y	Y	N

Questions	El-Ghazely et al., 2016	Esposito et al., 2018	Mayes et al., 2015	Mohseni et al., 2018	Twetman et al., 2018	Wälivaara et al., 2019	Tahir et al., 2014*
exposure or intervention of interest*							
8. Was follow-up complete and if not, were differences between groups in terms of their follow-up adequately described and analysed?	Y	Y	Y	Y	Y	Y	Y
9. Were participants analysed in the groups to which they were randomised?	Y	Y	Y	Y	Y	N	-
10. Were outcomes measured in the same way for treatment groups?/Were the outcomes of participants included in any comparisons measured in the same way?**	Y	Y	Y	Y	Y	Y	Y
11. Were outcomes measured in a reliable way?	Y	Y	Y	Y	Y	Y	Y
12. Was appropriate statistical analysis used?	Y	Y	Y	Y	Y	Y	Y
13. Was the trial design appropriate, and were any deviations from the standard RCT design (individual randomisation, parallel groups) accounted for in the conduct and analysis of the trial?	Y	Y	Y	Y	Y	Y	-

Questions	El-Ghazely et al., 2016	Esposito et al., 2018	Mayes et al., 2015	Mohseni et al., 2018	Twetman et al., 2018	Wälivaara et al., 2019	Tahir et al., 2014*
14. Is it clear in the study what is the 'cause' and what is the 'effect' (i.e. there is no confusion about which variable comes first)?**	-	-	-	-	-	-	Y
15. Was there a control group?**	-	-	-	-	-	-	Y
16. Were there multiple measurements of the outcome both pre and post the intervention/exposure?**	-	-	-	-	-	-	NA
% Yes	100	53.84	76.92	100	100	92.30	77.77

Y: Yes; N: No; U: Unclear; NA: Not Applicable; *similar questions on both instruments **Specific question from Critical Appraisal Checklist for Quasi-Experimental Studies (non-randomised experimental studies)
